# Review of Nipple Reconstruction Techniques and Introduction of V to Y Technique in a Bilateral Wise Pattern Mastectomy or Reduction Mammaplasty

**Published:** 2015-04-06

**Authors:** Charles A. Riccio, Matthew R. Zeiderman, Saeed Chowdhry, Bradon J. Wilhelmi

**Affiliations:** ^a^University of Louisville School of Medicine, Louisville, Kentucky; ^b^Division of Plastic Surgery, University of Louisville School of Medicine, Louisville, Kentucky

**Keywords:** nipple reconstruction, nipple areola complex, postmastectomy, V-Y, mammaplasty

## Abstract

**Introduction:** Nipple-areola complex reconstruction (NAR) is the final procedure in breast reconstruction after the majority of mastectomies. Many methods of NAR have been described, each with inherent advantages and disadvantages depending on local healthy tissue availability, previous scarring and procedures, and the operative morbidity of the NAR technique. Nipple reconstructions may be complicated by scars or previous nipple reconstruction, making the procedure more challenging. We propose the use of the V-Y advancement flap as a new method that is suitable for both novice and experienced surgeons wishing to broaden their range of techniques for difficult nipple reconstructions. **Methods:** A traditional V-Y advancement flap is lifted at the site of the future nipple. Mastectomy scars from prior mastectomy, mammoplasty, or nipple reconstruction can be incorporated into the flap. The flap is folded caudally upon itself and the secondary defect at the apex of the flap is linearly closed. **Results:** At 6-month postoperative evaluation, adequate nipple projection and patient satisfaction were achieved with this method. **Conclusion:** The V-Y advancement flap is a suitable method for achieving satisfactory results when faced with challenging NAR. The method is easy to perform, reproducible, has low operative morbidity, and incorporates previous wise pattern mastectomy or mammaplasty scars into the newly reconstructed nipple, thereby decreasing new scar formation on the breast and leading to favorable cosmetic results.

While the medical literature contains many technical descriptions regarding nipple reconstruction, very few randomized clinical trials exist to support an evidence-based consensus establishing which techniques are superior. Therefore, no definitive indications currently exist dictating when a certain technique should be employed.^1^ Nipple-areola complex reconstruction (NAR) is the final procedure in breast reconstruction after the majority of mastectomies and, in the opinion of many women, represents the defining feature of the female breast.^2^ Previous studies have demonstrated that timely completion of nipple and areola construction leads to improved psychological well-being in addition to patient and partner satisfaction.^3^^,^^4^ Nipple reconstructions may be complicated by scars or previous nipple reconstruction, making the procedure more challenging as they are randomly based flaps. For this reason, we propose the use of the V-Y advancement flap as a new method that is suitable for both novice and experienced surgeons wishing to enhance their scope of techniques for nipple reconstructions.

## METHODS

**Figure 1:**
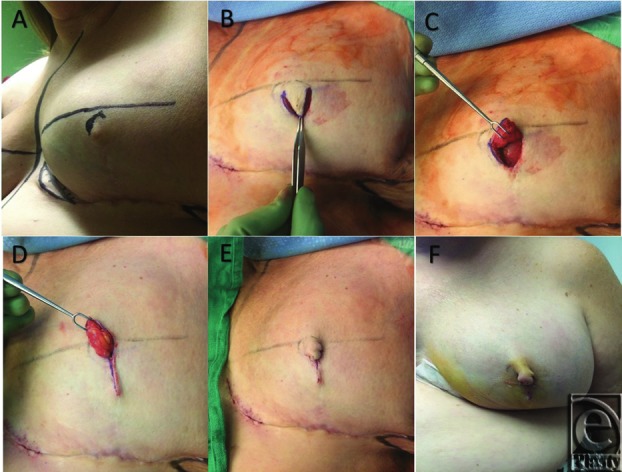
V-Y advancement flap for NAR: (A) preoperative markings. (B &C) Elevation of the flap with subdermal fatty pedicle. (D) Vertical advancement closure of nipple site. (E &F) Intraoperative and postoperative final results of V-Y NAR.

**Figure 2:**
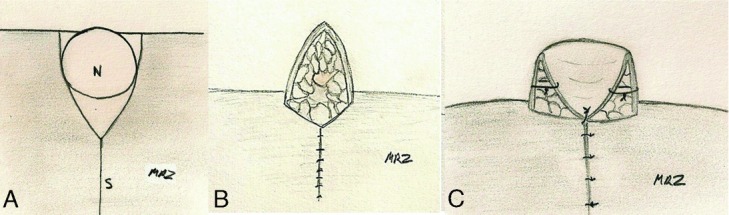
V-Y advancement flap. (A) Incision outlines including site of future nipple (N) a mastectomy scar (S) to be incorporated (B) elevation of flap with subdermal fatty pedicle and closure of V incisions (C) caudal folding of the flap, inferior view.

This patient had undergone mastectomy with wise pattern incisions. The initial closure created a dog ear at the desired nipple location. However, it flattened over time. For redo, the patient is marked in the upright position and the symmetrical placement of the new nipple site is chosen by the physician and the patient. The position of the new nipple is outlined and the base of the V incision is set at the superior border of the nipple (Figs 1A and 2A). The apex of the V is aligned to meet on the vertical scar at least 2 cm below the base of the V-flap in order to provide adequate nipple projection (Fig 1B). Incisions are made through the epidermis, dermis, and subcutaneous fat, and the flap is elevated by dissection in the deep subcutaneous plane (Figs 1C and 2B). The secondary defect at the apex of the flap is subsequently closed in a linear fashion with 3-0 PDS and 5-0 chromic sutures (Fig 1D). The apex of the flap is folded caudally upon itself and the edges are approximated with 5-0 chromic sutures (Figs 1E-1F and 2C).

## RESULTS

At a 6-month postoperative evaluation, the patient presented with a result satisfactory to both the patient and the surgeon. Visible new scar tissue on the breast was not increased because of incorporation of the previous mastectomy scar into the flap. Nipple projection was adequately achieved and maintained. The flap was based superiorly where there was no scar to optimize blood supply. The donor site closure did not flatten the breast or change breast shape.

## DISCUSSION

Despite the wide variety of local flap designs described in the scientific literature, several constants exist with regard to successful flap construction. These include a wide pedicle to preserve the existence of a reliable blood supply, the feasibility of employing the selected pattern and technique through simplicity of flap design, the separation from surrounding tissues to minimize centrifugal retraction forces, and the ability to use existing scars to achieve optimal cosmetic results.^5^ The literature indicates that the most popular method of NAR is the use of subcutaneous random pedicle flaps. These flaps are raised as full-thickness skin flaps and have gained popularity over central flaps because of their superiority in creating a long-lasting projection. It must be noted that all flaps are subject to some retraction as a result of the contracture of superficial scars, scarring of the flap itself, or irradiation. However, pedicle flaps are not subject to the same degree of retraction forces, especially in regard to those exerted by the surrounding and underlying tissues, which act on the entire base of central flaps. This is because the majority of the flap exists independently of the underlying tissue. Furthermore, pedicle flaps experience a comparatively better blood supply since the source is through the subdermal plexus rather than the limited supply afforded through the subcutaneous tissue on which central core flaps are dependent.^6^^,^^7^ Despite the numerous techniques described for achieving the purpose of NAR, the most popular utilize a single pedicle and include the skate flap, star flap, C-V flap, the double opposing periareolar and double opposing tab flaps, the diamond double opposing flap, as well as the mushroom flap.

The V-Y flap is a modified advancement flap with a local pedicle that allows for structure elongation and is commonly used for the closure of small to medium cutaneous defects of the face.^8^ We have modified this technique for the use of nipple reconstruction by using the base of the flap to form a new nipple while also incorporating the scar from a previous wise pattern mastectomy or reduction mammaplasty into the incision closure. Pertaining to this technique, our first patient was a white female who presented for redo nipple reconstruction due to loss of projection of her reconstructed nipple. The V-Y method of repair was ideal because of her deficiency of healthy, unscarred tissue as a result of previous mastectomy, and nipple reconstruction. The V-Y advancement flap was ideal in this case, and for any such case, where other well-known methods would be technically challenging or likely produce a poor result secondary to compromised blood flow through a preexisting scar. The V-Y technique is easy to use, provides a reliable vascular supply, has minimal operative morbidity, and incorporates old scar tissue from a wise pattern mastectomy into the reconstruction, thereby minimizing scar burden and creating an aesthetically pleasing result.

The base of the flap can also be oriented vertically, allowing incorporation of horizontal scars as well. When used with a horizontal base, the scars from advancement of the flap are concealed on the inferior surface of the nipple, resulting in a more aesthetically appealing result for the patient. Another advantage of V-Y flap technique is that it forms only 1 new scar, and integration of previous vertical mastectomy scars into the reconstructed nipple helps maintain long-term nipple projection due to decreased fat resorption from the remodeled, fibrotic scar tissue. As a subdermal pedicle flap, the V-Y is not subject to the same retraction forces as centrally based flaps, which helps maintain long-term projection. Linear closure of the small donor site prevents distortion and flattening of the breast tissue. The maintenance of the subdermal pedicle and subcutaneous fat decreases fat resorption and necrosis because the native vascular architecture is not disrupted. Furthermore, by including the subdermal fat in the newly reconstructed nipple, the use of cartilage or synthetic fillers can be avoided. This is desirable, as cartilage filler generally results in a firm, unnatural feeling nipple and carries a risk of complications such as extrusion, which requires subsequent revision or removal.

The V-Y flap donor site is closed primarily, thereby avoiding the need for a full-thickness skin graft, which can be complicated by graft ischemia and also requires the creation of a new donor site. The native skin of the breast is left intact. This makes areolar tattooing easier to perform and more likely to maintain long-term pigmentation, whereas donor grafts frequently taken from the inner thigh are less reliable for maintaining tattoo pigmentation.

In addition, the V-Y technique is a suitable method for redo reconstruction of a C-V or star flap that has lost projection while simultaneously minimizing the horizontal scar left from prior reconstruction. The V-Y incision can have the base oriented perpendicular to the horizontal scar of the C-V reconstruction, thereby incorporating and reducing scar tissue.

One of the earliest NAR techniques described in the literature is a mushroom pedicle-based flap. The basis for the technique rests in the postulation that the best way to ensure nipple protrusion is by making it difficult if not impossible for retraction, much in the same way as an irreducible hernia would behave. A circular split-thickness island of skin is created with the intention of preserving central circulation through a stalk. This represents approximately one quarter of the surface area of the constructed areola attached at the intended nipple site. To construct the new areola, a full-thickness skin graft is harvested from the area below the inguinal crease and sutured over the elevated nipple segment including its central island. Subsequently, a small cruciate incision is created centrally to permit protrusion of the flap on its stalk through the opening. This prevents the undersurface of the split-thickness skin flap from reattaching since its vascular bed is covered by a full-thickness graft.^6^^,^^7^

The skate flap was first described by Little in 1984 and has since become one of the most popular techniques. It can be safely performed over breast implants with predictable results and acceptably low rates of complications.^9^ This technique was demonstrated to maintain long-term stable nipple projection and volume compared to the bell-flap techniques, especially after 6 months.^10^ However, Zhong et al’s^9^ retrospective review of 422 patients who underwent NAR with the use of a “modified skate” technique found that long-term projection was only modest and patients should be forewarned of that eventuality before electing to undergo the procedure.^10^ In addition to the discrepancies in the literature regarding the long-term maintenance of results obtained through utilization of the skate flap technique, there are several other considerations before reconstruction by this method can be performed. The patient must have a flap-based skin island remaining from previous reconstructive procedures such as a latissimus dorsi or TRAM flap, or adequate vascularity to the skin must exist from impressively thick flaps originating from the original mastectomy. The procedure involves elevation of a significantly vascularized and voluminous vertical cutaneous fat flap to serve as the fat core. Subsequently, two split-thickness wings are then wrapped around this core to create the reconstructed nipple. As a variation, Hammond et al have since described using a purse string closure of the areolar complex to the breast tissue, thereby closing the donor defect for the reconstructed areola by primary intention without the use of an autologous skin graft.^11^^,^^12^ Shestak and colleagues described a very similar technique termed the double-opposing periareolar flap, which also relies on a derivative of the skate flap and closes the defect by primary intention with a purse string suture closure.^13^ The techniques described by Hammond and Shestak offer the unique advantage of containing all donor site scars within the peripheral periareolar incision. Areolar tattooing following the procedure allows for masking of the scars.

A similar technique that has also gained popularity is the star flap. This technique is ideally used for nipple reconstruction at least 3 months after implant-based breast reconstruction where a preexisting mastectomy scar passes through the intended site of new nipple.^14^ The major difference between the skate and star flaps is that in the star flap, both skin and subcutaneous tissue are raised in the wings, instead of simply the dermis, allowing for primary closure of the donor area. The advantage of this technique is that the mastectomy scar can be used in the flap design with an ideally located scar, enabling optimal positioning of the nipple and improved cosmetic outcomes.^14^ However, if the horizontal scar is not ideally located, the resulting nipple may be located too far superiorly or inferiorly. In addition, this technique intrinsically creates an unappealing vertical scar upon elevation of the central limb of the flap. Moreover, incorporation of the scar into the central limb of the flap predisposes to nipple necrosis because the central limb is based on the scar. Finally, like the C-V flap, the size of the reconstructed nipple is limited by the wing length of the flap, and the long-term aesthetic appearance of nipple projection can be unreliable as a result of absorption of the central fat core.^15^

Yet another option for incorporating the NAR into a preexisting mastectomy scar is the double-opposing flap described by Kroll et al in 1989.^16^^,^^17^ In this technique, 2 opposing dermal pedicle flaps are elevated with tab extensions similar to those described by Little in the skate flap technique. The flap donor sites are then closed by suture approximation allowing the flaps to be advanced centrally in opposition, where they can then be used to support each other's projection. No skin graft is required on the nipple itself since the flaps cover all exposed fat. This technique is ideal for reconstruction with a tissue expander or implant because it places the nipple at the center of the scar, all scars are contained within the incision sites, the resultant nipple is located where it was premastectomy, and long-term nipple projection appears to be consistent with that expected with the skate-flap technique.^10^^,^^17^

The C-V flap was developed in 1998 and has since established a long track record in regard to successful nipple reconstructions and patient satisfaction.^18^^,^^19^ In contrast to the skate flap technique, 2 lateral “V” shaped flaps are elevated at the subcutaneous level, a “C” shaped dermal flap is elevated centrally, and the donor sites are subsequently closed. The V flaps are then interwrapped with the C flap secured on top to act as the cover.^20^ Despite issues with maintaining long-term nipple projection, in a 2007 study by Eo and colleagues,^21^ it was demonstrated that improved projection can be achieved with the addition of an autologous dermal fat graft, which can be obtained from excess tissue present at mound revision.^19^ However, as stated previously, nipple size is limited by flap dimensions, and if a nonideally placed scar is present, the resultant nipple will be inappropriately located from the natural anatomic site.

In 2010, Lesavoy and Liu^22^ described the diamond double-opposing V-Y flap. In this technique, the diamond skin flap is centered over new nipple position, and full-thickness incisions are made through the skin and dermis. The lateral and medial thirds of the limbs are undermined elevated and the central third is left undisturbed to serve as the subcutaneous pedicle. The medial and lateral horizontal flaps are transposed and sutured together on the inferior aspect of nipple, thereby forming the new nipple. The diamond-shaped donor wound is subsequently closed in the typical V-Y fashion. This technique is easy to perform, allows incorporation of previous horizontal mastectomy scars into the newly reconstructed nipple, and maintains good long-term projection. However, if prior mastectomy scars are not present or cannot be incorporated into the flap, this method results in the creation of 2 new scars. Furthermore, this technique cannot be oriented vertically, as it will place the scar in the upper pole of the breast. This is undesirable since it is visible in certain outfits or swimsuits.

## CONCLUSION

The V-Y advancement flap for NAR is a novel, easy-to-perform method that is useful for breasts with vertical or wise pattern scars as well as for redo nipple. The technique provides a reliable vascular supply to the new nipple, has minimal operative morbidity, and minimizes scar burden by raising a flap with preserved blood supply and no scar across its base, yielding an aesthetically pleasing result. Additional advantages include avoiding skin graft donor site morbidity, as well as preserving skin for more reliable tattooing and avoiding foreign body fillers. Our technique offers another option to the armament of the reconstructive surgeon dealing with technically challenging nipple areola complex reconstructions in breasts with variant scars.
